# Phantom Plague: How Tuberculosis Shaped History

**DOI:** 10.3201/eid2903.221676

**Published:** 2023-03

**Authors:** Henry M. Blumberg

**Affiliations:** Emory University School of Medicine and Rollins School of Public Health, Atlanta, Georgia, USA

**Keywords:** tuberculosis and other mycobacteria, antimicrobial resistance, bacteria, respiratory infections, drug-resistant tuberculosis, India, book review

Although the subtitle of Phantom Plague by Vidya Krishnan is How Tuberculosis Shaped History, the book is actually about how history shaped tuberculosis (TB) and how TB transmission and disease has surged and persisted because of conditions such as poverty, crowding, a lack of political commitment, and poor public policies (Figure). As noted in the book, “poverty is the disease, TB the symptom.” The author focuses largely on the TB epidemic in India, especially in Mumbai, India, and provides heartbreaking narratives of several persons with TB, including Shreya Tripathi, who had drug-resistant TB and whose life inspired the book. The book is an indictment of the healthcare system in India, care provided to patients with TB (often by private providers) in India and elsewhere, and TB control and prevention program bureaucracy, notably India’s current government efforts and a somewhat farcical declaration that TB would be ended in India by 2025. 

**Figure:**
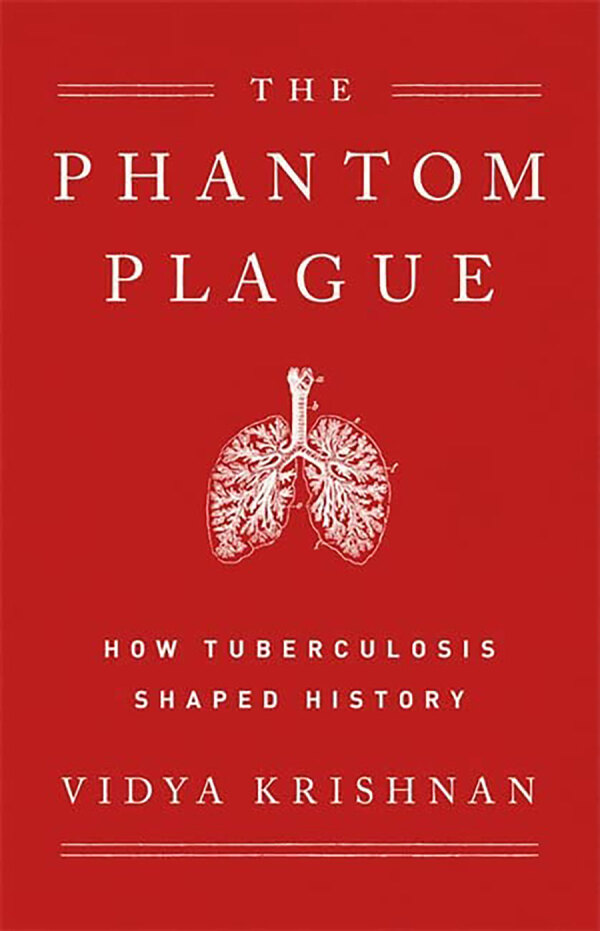
Phantom Plague: How Tuberculosis Shaped History

The book also singles out “the rise of the Hindu supremacist movement [in India which] has brought with it a tsunami of misinformation and science denialism”; misinformation and science denialism have been similarly unleashed in the United States during the COVID-19 pandemic. The author highlights many challenges ahead that limit achieving the World Health Organization (WHO) End TB strategy without substantial additional investments and development of new tools to combat TB (the WHO End TB strategy targets a 90% reduction in TB cases and 95% reduction in TB-related deaths by 2035). The book is titled Phantom Plague because >30% of global TB cases are never detected, highlighting the urgent need for simple, effective point-of-care TB testing. TB case detection has only worsened during the COVID-19 pandemic, as outlined in the recent WHO Global Tuberculosis Report 2022 (*1*). 

The book follows several tangential threads to provide some historical perspective on pandemics. The author, a writer and journalist based in Goa, India, uses those analogies to make her case for the problems and challenges in diagnosing and treating patients, especially those with drug-resistant TB. She takes the reader on journeys to discover how TB was viewed in the late 19th century in America and how historical figures, such as Semmelweis, Pasteur, and Koch, dealt with other challenges and epidemics (and how Koch dealt with TB specifically). Despite being an enjoyable read and journey, several small errors exist that someone with a science or medical background, especially in TB, might find pestiferous and could have been corrected by further editorial review. For example, dexamethasone (a steroid drug) is listed as an antibiotic, efficacies of bedaquiline and delamanid are likely not equivalent as claimed, and patients with drug-resistant TB are not treated with 10–15 drugs/day, although a substantial number of pills might be required.

The author also tackles racism issues suggested by the lack of an adequate global response to TB. She indicates that her book “is an attempt to show how often social misery inflicted on black and brown nations is quantified into ‘targets’” that never reflect the pain and suffering experienced by Shreya and others with drug-resistant TB. In addition to India’s government, the book is critical of a whole host of entities that include the “West [that] is simply unable to reimagine global health without a role for itself as the savior,” other governments, WHO’s stewardship of TB, and “Big Pharma, Big Tech, and Big Philanthropy.” The author criticizes the Gates Foundation, in part because of Bill Gates’ strong support for intellectual property laws. A considerable portion of the book is spent criticizing patent laws, which the author asserts are responsible for restricting availability of newer drugs in low- and middle-income countries where TB is most prevalent. What is not discussed or emphasized in the book is the lack of new drug development for TB treatment. In the past 35 years, 58 new drugs or drug combination have been approved by the US Food and Drug Administration for HIV treatment, yet only 2 new TB drugs have received approval (bedaquiline and pretomanid; pretomanid in conjunction with bedaquiline and linezolid as part of the BPaL regimen). The market forces that support drug development have not been conducive for TB because 99.9% of TB cases occur outside the United States (mainly in low- and middle-income countries with high TB prevalence), resulting in sparse interest by the pharmaceutical industry in developing new TB drugs. New models to support drug development for TB are urgently needed, but this issue is not discussed in the book. Finally, perhaps the book ends prematurely, because after it was written, new treatments were developed for highly drug-resistant TB that shifted to all oral regimens (WHO recommendation); a ≈90% favorable outcome was recently reported for the BPaL regimen used to treat highly drug-resistant TB (*2*). 

In summary, despite some flaws, this book is an interesting, easy read about some of the many challenges on the long road ahead toward the ultimate goal of TB elimination. The book also reminds us of the importance of community engagement: “listening to the voices of the affected community in the development and implementation of treatment options for drug-resistant tuberculosis is paramount” (*3*).
